# Reduction of Visual Artifacts in Laser Beam Scanning Displays

**DOI:** 10.3390/mi16080949

**Published:** 2025-08-19

**Authors:** Peng Zhou, Huijun Yu, Xiaoguang Li, Wenjiang Shen, Dongmin Wu

**Affiliations:** 1 School of Nano-Tech and Nano-Bionics, University of Science and Technology of China, Hefei 230026, China; pzhou2015@sinano.ac.cn; 2 Suzhou Institute of Nano-Tech and Nano-Bionics, Chinese Academy of Sciences, Suzhou 215123, China

**Keywords:** MEMS mirror, raster scanning display, banding artifacts, non-uniform distribution

## Abstract

Laser beam scanning (LBS) projection systems based on MEMS micromirrors offer advantages such as compact size, low power consumption, and vivid color performance, making them well suited for applications like AR glasses and portable projectors. Among various scanning methods, raster scanning is widely adopted; however, it suffers from artifacts such as dark bands between adjacent scanning lines and non-uniform distribution of the scanning trajectory relative to the original image. These issues degrade the overall viewing experience. In this study, we address these problems by introducing random variations to the slow-axis driving signal to alter the vertical offset of the scanning trajectories between different scan cycles. The variation is defined as an integer multiple of 1/8 of the fast-axis scanning period ($1/f_{h}$) Due to the temporal integration effect of human vision, trajectories from different cycles overlap, thereby enhancing the scanning fill factor relative to the target image area. The simulation and experimental results demonstrate that the maximum ratio of non-uniform line spacing is reduced from 7:1 to 1:1, and the modulation of the scanned display image is reduced to 0.0006—below the human eye’s contrast threshold of 0.0039 under the given experimental conditions. This method effectively addresses scanning display artifacts without requiring additional hardware modifications.

## 1. Introduction

Micro-electro-mechanical systems (MEMS) mirrors are miniature optical scanners designed to generate time-varying angular deflections of light for beam-steering applications. In 1980, Kurt Petersen introduced the potential use of silicon micromachining for fabricating scanning mirrors [1,2]. Since then, MEMS mirrors have been widely applied in various fields, including barcode readers, video displays, laser printers, LIDAR, and so on [2,3,4,5,6,7,8]. These systems, referred to as laser beam scanning systems, utilize MEMS mirrors and optics to generate a flying spot that creates a two-dimensional scanning trajectory. During scanning, the beam undergoes ultra-high-frequency modulation, which enables single-pixel information adjustment to realize system functions [9,10,11,12].

**Figure 1:**
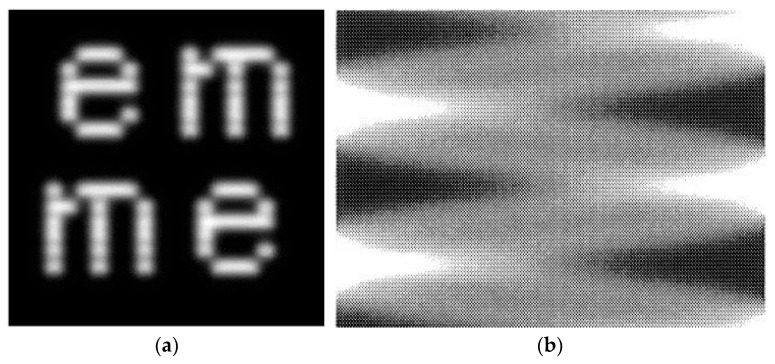
(**a**) Banding artifacts; (**b**) non-uniform line spacing artifact.

Raster scanning is a widely used beam scanning technique, where the beam performs sinusoidal motion in the horizontal direction and linear motion in the vertical direction [13,14,15,16]. This scanning method provides good trajectory stability and higher scanning density; therefore, some laser scanning display products are based on this technology. However, one significant artifact observed in raster scanning images is the appearance of banding artifacts between horizontal scan lines, introducing periodic noise into the projected image [9,14,17,18]. As shown in Figure 1a, the display system presents an image of the word “emme”. However, banding artifacts can be observed above the characters, and the displayed word appears neither smooth nor continuous. Another artifact is the non-uniform line spacing in the case of bidirectional scanning, leading to inconsistent vertical pixel spacing, with the most pronounced variation occurring at the edges of the scanning trajectory, where more noticeable banding artifacts also tend to appear, as shown in Figure 1b. These artifacts degrade image quality and may result in display inaccuracies.

The phenomenon of banding artifacts was discussed in Articles 1 and 9. Given the human eye’s high sensitivity to periodic patterns, the luminance variation in the vertical direction of the scanning pattern becomes highly perceptible. However, this article only provided the optimized projected image, while the implementation details for achieving this optimization remained unspecified. Regarding the non-uniform spacing artifact, Articles 13 and 15 described it as the “Raster Pinch” effect, where the forward and return half-period raster lines are “pinched” near the edges of the scanning image, resulting in noticeable distortion in the vertical pixel spacing at the image boundaries. They proposed that a third scanner could be added to the system to create an approximation to a staircase motion in the vertical axis and correct for the non-uniform raster spacing. Nonetheless, this solution increases the overall system complexity.

In this paper, we propose a novel scanning projection method to mitigate both the banding artifacts and the “Raster Pinch” effect. The design of this method takes into account the temporal characteristics of human visual perception: typical projection refresh rates are around 60 Hz, corresponding to about 16 ms per frame, whereas the human eye has an integration time of approximately 30 ms—roughly equivalent to two frame durations. Leveraging this relationship, we introduce random variations into the scanning trajectories so that the path in each frame differs from those of other frames and covers regions that were not scanned. By visually integrating consecutive frames with different scanning trajectories, the human eye perceives a scanning display image with a higher fill factor. During the projection process, the image corresponding to each scanning trajectory is recalculated based on its actual position relative to the ideal rectangular image. As a result, the projected image achieves higher accuracy and improved visual quality.

The rest of this paper is organized as follows: Section 2 explores the underlying causes of the dark band phenomenon and non-uniform spacing. Section 3 outlines the method for adjusting the scanning trajectory. Section 4 presents the experimental results.

## 2. Raster Pinch Effect and Banding Artifacts

### 2.1. Raster Pinch Effect

**Figure 2:**
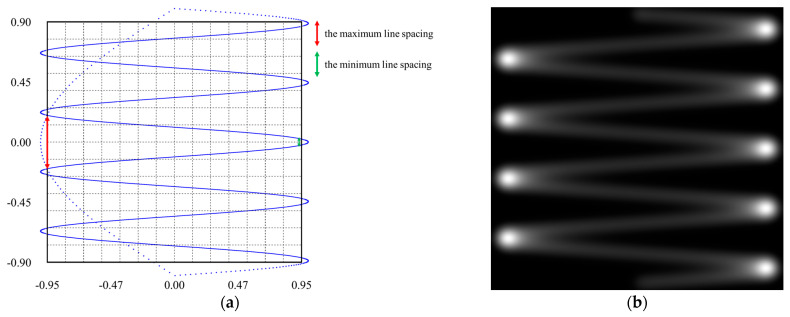
(**a**) Schematic diagram of the raster scanning trajectory with horizontal bidirectional sinusoidal motion and vertical linear motion; (**b**) intensity distribution along the scanning trajectory of a Gaussian beam.

The Raster Pinch effect refers to the non-uniform distribution of the scanning trajectory in the vertical direction. The raster scanning trajectory is formed by a spot performing a sinusoidal reciprocating motion in the horizontal direction while concurrently moving linearly downward in the vertical direction. Upon reaching the bottom position, the spot rapidly returns to the initial top position, completing a full scanning cycle [19]. This trajectory is consistently repeated in subsequent frames. During the spot’s return to the starting position, the light is turned off. A simple 2D raster scan trajectory is illustrated in Figure 2a. The horizontal scanning is a sinusoidal motion at 100 Hz, while the vertical scanning follows a 20 Hz triangular wave with a duty cycle of 9:1.

In the scan trajectory, the effective region for displaying a rectangular image is defined within the solid-line box, where the laser remains “on”, while it is “off” outside this boundary. The laser is turned off on the left and right sides due to excessive brightness in these areas, caused by the spot slowing down. This phenomenon is illustrated in Figure 2b, which depicts a Gaussian spot scanning along the forward trajectory (from top to bottom). Additionally, the laser is off at the top and bottom areas to maintain the rectangular integrity of the trajectory and provide a margin for system adjustments, such as the alignment of laser spots with different wavelengths.

The grid intersection points in Figure 2a represent the image ideal pixel positions to be projected. Analyzing the scan trajectory within the effective region reveals that, due to the bidirectional scanning characteristics, the spacing between adjacent scan lines varies. On one horizontal side, the line-to-line spacing is reduced to less than half of the pixel pitch (the red arrows in Figure 2a), whereas on the opposite side, it expands to approximately three times the pixel pitch (the green arrows in Figure 2a). In cases where the spacing exceeds the pixel pitch, some image pixels may not be displayed, leading to missing image information. To preserve the completeness of the projected image, it is essential to address the problem of non-uniform scan line distribution. In addition, this non-uniform distribution contributes significantly to the formation of banding artifacts near the left or right edges of the scanned image.

### 2.2. Banding Artifacts

When observing unoptimized scanning projection images, banding artifacts appear as vertically aligned dark bands overlaid on the original image, introducing unwanted visual interference. The spacing between bands corresponds to the inter-line spacing of the scanning trajectory and is closely related to the image resolution. Resolution is a key criterion for evaluating image quality and it depends on multiple factors, including the horizontal frequency $f_{h}$ of the MEMS mirror, vertical scanning frequency $f_{v}$, mirror aperture *D*, optical deflection angle θ, laser wavelength λ, and laser modulation frequency $f_{\mathrm{LD}}$. Notably, the vertical scanning frequency $f_{v}$ corresponds to the refresh rate of the image, with a typical value being 60 Hz.

Assuming a small optical deflection angle θ (paraxial approximation), and based on the mirror aperture *D* and laser wavelength λ, the number of resolvable spots along the horizontal or vertical direction *N* can be written as [2]

(1) $$N=\;\frac{\theta D}{a\lambda }\;,$$

where a (typically between 0.75 and 2) is a shape factor determined by the definition of spot size (amount of overlap between adjacent spots), mirror shape, angle of incidence, and beam profile. This formula indicates that the optical resolution, i.e., the number of resolvable spots, can be obtained by dividing the total optical deflection angle θ by the diffraction-limited angular resolution aλ/D. In some studies, the pixel size is typically chosen as equal to full width at 50% irradiance of the spot (FWHM) [20].

**Figure 3:**
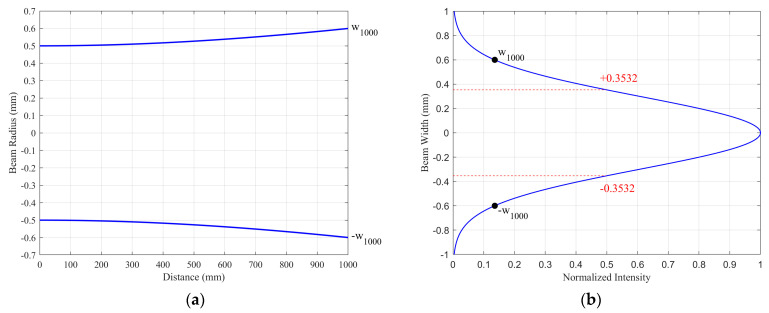
(**a**) Divergence of a Gaussian beam with a waist radius of 0.5 mm at the position of the MEMS mirror, with the screen approximately 1000 mm away; (**b**) intensity distribution of the spot at the screen.

Lasers are employed as the light source in scanning display systems due to the excellent collimation, enabling clear image projection over a wide range of distances. The intensity distribution of the laser spot can be approximated as a rotationally symmetric Gaussian beam (M^2^ = 1), which diverges over distance according to the following equation [21]:

(2) $$w^{2}\left(z\right)\;=\;w_{0}^{2}\left[1+{\left(\frac{\lambda z}{\pi w_{0}^{2}}\right)}^{2}\right],$$

where *z* denotes the axial distance along the beam’s propagation direction, measured from the beam waist position (*z* = 0), where the beam radius is at its minimum. $w_{0}$ is the beam waist radius at which the intensity drops to 1/e^2^ of its maximum value and w(z) is the beam radius at a distance of z. In scanning display systems, the beam waist is commonly positioned at the MEMS mirror to maximize optical efficiency. Figure 3a illustrates the propagation of a Gaussian beam with a waist radius of 0.5 mm, located at the MEMS mirror, to a screen positioned 1000 mm away. The intensity distribution of a Gaussian beam is expressed as [22]

(3) $$I(r)=I_{0}e^{\left(-\frac{2r^{2}}{w^{2}(z)}\right)},$$

where $I_{0}$ is the peak intensity at the center of the beam, *r* is the radial distance away from the axis, and *w*(*z*) is the radius of the laser beam where the intensity is 1/e^2^ of $I_{0}$. The beam spot radius on the screen is 0.6 mm (denoted as w_1000_), and the full width at half maximum (FWHM) of the intensity profile, corresponding to the distance between the two red dashed lines, is 0.7064 mm, as illustrated in Figure 3b. Consequently, the inter-line spacing is set to this value.

**Figure 4:**
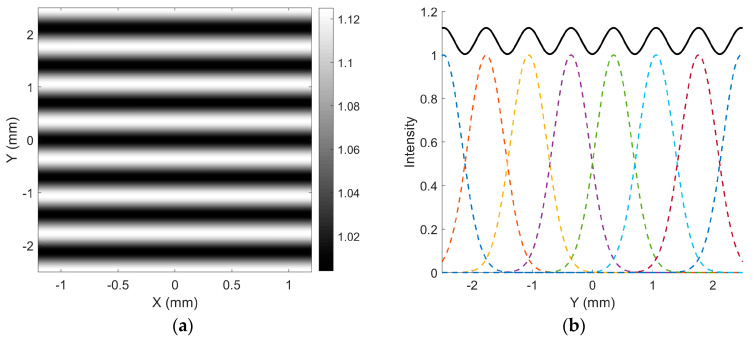
(**a**) Vertical distribution of eight Gaussian lines with spacing equal to the FWHM; (**b**) intensity profile of the eight Gaussian lines.

In the scanning trajectory, since the horizontal motion is significantly faster than the vertical motion, the scan lines in the central region of the image can be approximately considered parallel. Figure 4a illustrates a vertical scanning trajectory composed of eight parallel lines, separated by 0.7064 mm, corresponding to the FWHM of the beam intensity. The modulation *M* of the combined intensity is [21]

(4) $$M=\frac{I_{\max}-I_{\min}}{I_{\max}+I_{\min}}=\frac{1.125-1}{1.125+1}=0.059,$$

where $I_{\max}$ is the maximum intensity and $I_{\min}$ is the minimum intensity.

The spatial intensity variation between lines resembles a wave-like pattern, as shown in Figure 4b. When the source image is purely white, the resulting scanned image visually appears as a vertically distributed grating pattern.

Although the modulation between bright and dark areas is low, this phenomenon remains perceptible due to the human eye’s high sensitivity to periodic patterns. This sensitivity is characterized by contrast sensitivity, which is defined as the inverse of the modulation threshold of a sinusoidal luminance pattern. The modulation threshold of this pattern is generally defined by 50% probability of detection. A typical model was published by Barten in 1999 and the model results in the following equation for the contrast sensitivity function (CSF) [9,23,24]:

(5) $$S\left(u,L\right)=\frac{1}{m_{t}\left(u,L\right)}=\frac{M_{\mathrm{opt}}\left(u,L\right)/k}{\sqrt{\frac{2}{T}\left(\frac{1}{X_{0}^{2}}+\frac{1}{X_{\max}^{2}}+\frac{u^{2}}{N_{\max}^{2}}\right)\left(\frac{1}{\eta \mathrm{pE}}+\frac{\Phi_{0}}{1-e^{-{\left(u/u_{0}\right)}^{2}}}\right)}}$$

where *S* is the contrast sensitivity, $m_{t}$ is the modulation threshold, u is the spatial frequency, L is the luminance, $M_{\mathrm{opt}}\left(u,L\right)$ is the optical Modulation Transfer Function (MTF) of the eye, k is the signal to noise ratio, T is the integration time of the eye, $X_{0}$ is the angular size of the object, $X_{\max}$ is the maximum angular size of the integration area of the noise, $N_{\max}$ is the maximum number of cycles over which the eye can integrate the information, η is the quantum efficiency of the eye, E is the retinal illuminance in Troland, p is the photon conversion factor, which gives the number of photons per second per square degree per Troland, $\Phi_{0}$ is the spectral density of the neural noise, and $u_{0}$ is the spatial frequency above which the lateral inhibition ceases. By using the typical values of these constants, the equation reduces to [24]

(6) $$S\left(u,L\right)=\frac{1}{m_{t}\left(u,L\right)}=\frac{5200\;e^{-0.0016\;u^{2}\;{\left(1+100/L\right)}^{0.08}}}{\sqrt{\left(1+\frac{144}{X_{0}^{2}}+0.64u^{2}\right)\left(\frac{63}{L^{0.83}}+\frac{1}{1-e^{-0.02u^{2}}}\right)}}\;$$

**Figure 5:**
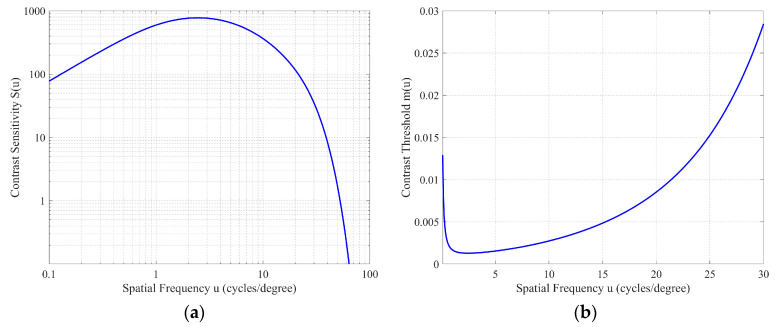
(**a**) Human eye contrast sensitivity function; (**b**) human eye contrast threshold.

Figure 5a illustrates the Contrast Sensitivity Function (CSF) curve based on a luminance of 100 cd/m^2^ and a field size of 30 × 30 degrees.

As stated above, the screen is positioned 1000 mm away, with line spacing of 0.7064 mm, resulting in a spatial frequency of 25 cycles per degree. At this spatial frequency, the threshold ($m_{t}\left(25,100\right)\;=\;\frac{1}{S\left(25,100\right)}$) is 0.017 (Figure 5b), while the modulation of the scanned image (Figure 4a) is 0.059, significantly exceeding this threshold. Therefore, observers can clearly perceive the banding artifacts.

## 3. Optimization Strategies

In accordance with the characteristics of human vision, suppressing banding artifacts requires either directly reducing modulation or increasing spatial frequency, as a higher spatial frequency increases the modulation threshold and makes the artifacts less perceptible. However, due to the aperture limitations of the MEMS mirror, increasing the spatial frequency by reducing the spot size is not feasible. Reducing modulation can be achieved by decreasing the line spacing, but this approach may compromise image clarity. Therefore, we propose an indirect method of modulation reduction: when the scanning spot of the second frame traverses the dark regions of the first frame, the human eye’s temporal integration enhances the luminance in these regions, thereby lowering the overall modulation and reducing the visibility of banding artifacts.

To realize this function, we modify the scanning trajectory of each frame by adjusting the retrace time within the scanning cycle. This adjustment causes the starting point of each frame to shift accordingly, while the top-to-bottom scanning duration must remain constant to maintain the number of vertical scanning lines, which corresponds to the image’s vertical resolution. This approach will inherently change the scanning time per frame, but the adjustment of the retrace time is bidirectional—allowing both increases and decreases—which enables the overall scanning cycle to remain relatively stable, thereby preventing image frame loss.

**Figure 6:**
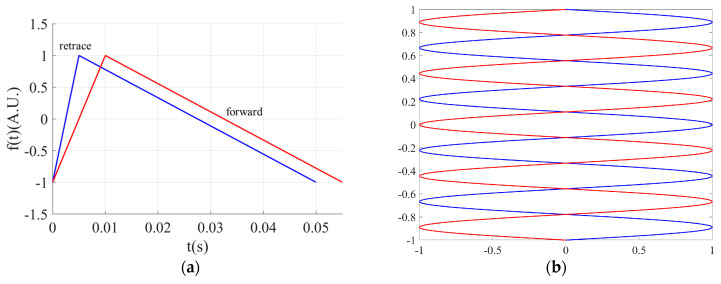
(**a**) Illustrated diagram of the initial drive waveform shown in blue and the modified drive waveform shown in red; (**b**) illustrated diagram of the initial scanning trajectory shown in blue and the modified scanning trajectory shown in red.

Adjusting the retrace time involves modifying the vertical drive signal of the MEMS mirror, represented by a triangular waveform, as illustrated in Figure 6a. The blue waveform represents an unmodified vertical drive signal with a scanning frequency of 20 Hz, which is defined as

(7) $$\;f(t)=\left\{\begin{array}{c} -1+\frac{2}{0.1\times \left(\frac{1}{20}\right)}t,\;\;0\;s\;\leq \;t\;\leq \;0.005\;s\\ -\frac{2}{0.9\times \left(\frac{1}{20}\right)}t+\frac{11}{9},\;\;0.005\;s\;<\;t\;\leq \;0.05\;s \end{array}\right.\;,$$

where *t* is the time variable of the driving signal, and f(t) is the customized amplitude of the driving signal. The red waveform corresponds to the drive signal after the retrace time has been modified by 0.005 s, defined as

(8) $$\;f(t)=\left\{\begin{array}{c} -1+\frac{2}{0.1\times \left(\frac{1}{20}\right)+0.005}t,\;\;0\;s\;\leq \;t\;\leq \;(0.005+0.005)\;s\\ \frac{-2}{0.9\times \left(\frac{1}{20}\right)}t+\frac{13}{9},\;\;\left(0.005+0.005\right)\;s\;<\;t\;\leq \;(0.005+0.05)\;s \end{array}\right.\;$$

The forward scanning trajectories for display, before and after adjustment, are shown in Figure 6b. The scanning trajectory is consistent with that in Figure 2, with horizontal scanning as a 100 Hz sinusoidal motion and vertical scanning as a 20 Hz linear motion. The vertical scanning trajectories can also be described by Equations (7) and (8). It can be observed that, after combining the trajectories of two successive frames, the fill factor of the scanning image relative to the original image is significantly improved, effectively suppressing the non-uniform distribution.

In this research, we found that applying only a single variation to the scanning trajectory is insufficient to eliminate banding artifacts, as the variation remains periodic, leading to the perception of flowing bands by the human eye. The scanning projection control system is implemented based on Field-Programmable Gate Array (FPGA). According to its working mechanism, the number of applied variables needs to be a power of 2. Therefore, we determine 16 variables $\left|\Delta t\right|$, with the minimum variable being 1/8 of the horizontal scanning period. The other variables are integer multiples of this value. The complete list of variables is as follows:

- ±1/8 of the horizontal scanning period (1/f_h_);
- ±2/8 of the horizontal scanning period (1/f_h_);
- ±3/8 of the horizontal scanning period (1/f_h_);
- ±4/8 of the horizontal scanning period (1/f_h_);
- ±5/8 of the horizontal scanning period (1/f_h_);
- ±6/8 of the horizontal scanning period (1/f_h_);
- ±7/8 of the horizontal scanning period (1/f_h_);
- 0 and −8/8 of the horizontal scanning period (1/f_h_).

**Figure 7:**
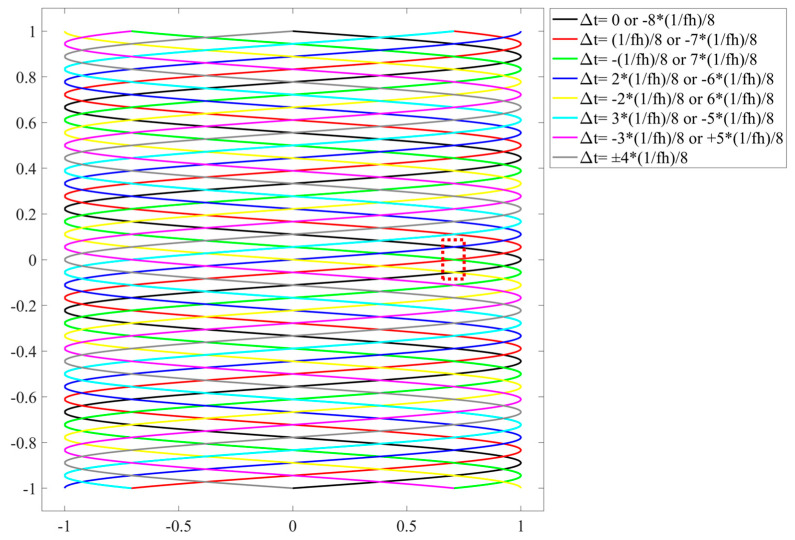
Scanning trajectories corresponding to 16 variables applied to the retrace time.

Figure 7 illustrates the differences in scanning trajectories under varying retrace time, where the trajectories coincide when the sum of two $\left|\Delta t\right|$ values is an integer multiple of the horizontal scanning period ($1/f_{h}$), as indicated in the legend. The black trajectory represents the initial state, and all these variables are relative to the initial state for the convenience of theoretical analysis. In practical control, the random variations are always applied to the previous signal rather than the initial state, resulting in continuous variations in the scanning trajectory without periodicity.

**Figure 8:**
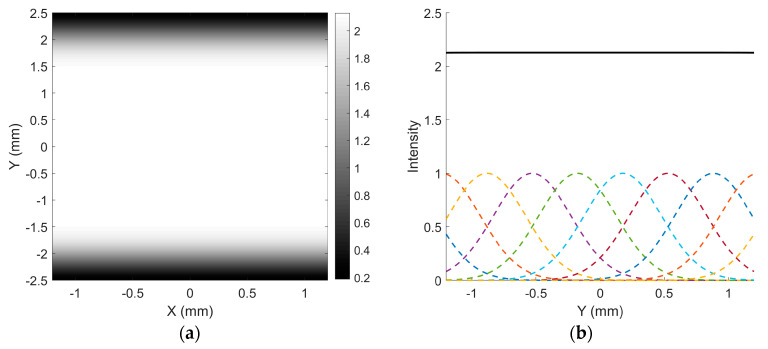
(**a**) Vertical intensity distribution of ten Gaussian lines with line spacing at 1/2 FWHM; (**b**) intensity profile of the central region of the ten Gaussian lines.

Taking the black trajectory as a reference in Figure 7, the red dashed box highlights the area where the fewest additional scanning lines are inserted between two adjacent lines due to the cross-overlapping of trajectories. As a result, the combined trajectory line spacing is reduced to half of the original spacing. Based on this, the intensity distribution of 10 Gaussian lines with half the spacing used in Figure 4 is illustrated in Figure 8a. As shown, the intensity within the central region comprising eight lines remains largely uniform, exhibiting no noticeable “waves”, and the modulation approaches zero, as seen in Figure 8b. In summary, by implementing the superposition of interleaved trajectories with different scanning periods during the eye’s integration time, the phenomenon of banding artifacts can be effectively mitigated.

**Figure 9:**
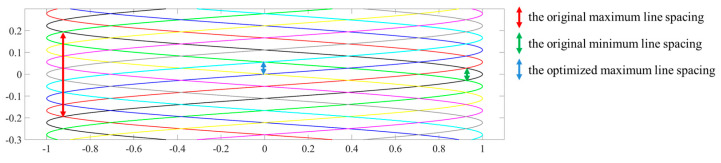
Illustration of the improved non-uniform distribution after optimization.

For the non-uniform spacing artifact, as indicated by the red and green arrows in Figure 9 (the middle region of Figure 7), the width on the left side is seven times that on the right. After interleaving multiple trajectories, the maximum line spacing in the composite trajectory (indicated by the blue arrow) matches that of the right-side green arrow. When projecting a static image, this significantly improves the non-uniform distribution. However, during video projection, the trajectory of each frame still exhibits non-uniformity. During projecting, the control system subdivides each line trajectory vertically and applies an interpolation algorithm based on the spot’s relative position between adjacent lines. This ensures accurate pixel color information and preserves scanning image fidelity.

## 4. Experimental Validation

**Figure 10:**
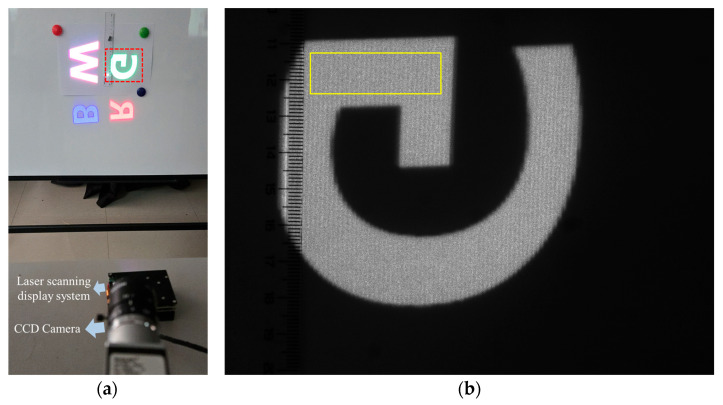
(**a**) Laser scanning projection system; (**b**) Image of the area indicated by the red dashed box in (**a**), captured by the CCD camera.

A laser projection system was established with a MEMS mirror featuring a horizontal frequency of 14.6 kHz and a vertical frequency of 60 Hz, achieving an image resolution of 640 × 400 pixels. RGB primary color lasers were employed, and scanning images on the screen were captured using a CCD camera (Basler acA 1600-60 gm, Basler AG, Ahrensburg, Germany) equipped with a 50 mm focal length imaging lens. The screen was positioned approximately 700 mm away from the scanning projection system, and the CCD camera was placed 1 m away from the screen. The entire experimental system and the scanning image are shown in Figure 10. During the testing process, the scanning projection system was rotated by 90 degrees, resulting in the bands in the scanning trajectory being arranged vertically.

The human eye is most sensitive to green; therefore, in this study, we primarily observed and analyzed the green regions of the image shown in Figure 10b. The screen used common printing paper, an easily accessible medium that helps suppress the laser speckle effect. In addition, a ruler was placed on the left side of Figure 10b as a reference to evaluate the periodicity of the banding artifacts. We can convert the number of pixels in the image to spatial units in millimeters based on the ruler scale. Then, using trigonometry and the known distance between the camera and the screen, we convert the image’s intrinsic frequency from cycles per pixel to spatial frequency units of cycles per degree.

**Figure 11:**
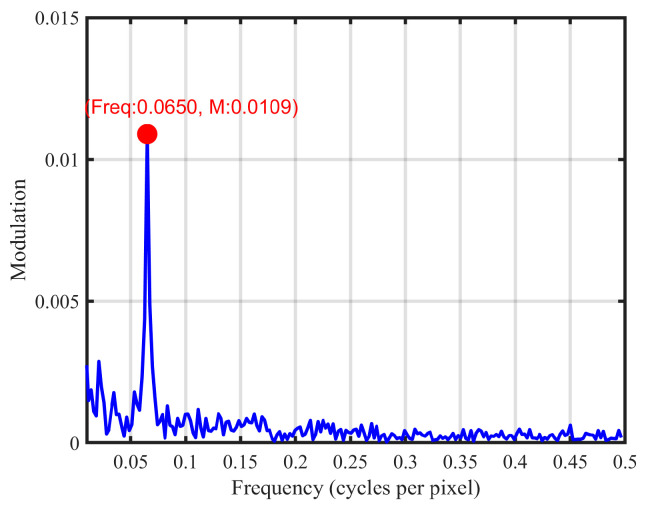
Spatial frequency profiles extracted from the yellow rectangular region in the single-frame scanning trajectory.

As previously mentioned, within a single scanning cycle, the trajectory itself still exhibits these artifacts. The image in Figure 10b was captured with an exposure time of one frame period (1/60 s), corresponding to a setting of 16,666 μs in the CCD camera control system, and a yellow rectangular region was selected for analysis. To reduce the impact of speckle noise, vertical averaging was applied, followed by a fast Fourier transform (FFT) to obtain the horizontal spatial frequency profiles shown in Figure 11 [9,25]. The analysis revealed a period of 15.4 pixels per cycle, corresponding to a spatial frequency of 13.6 cycles per degree, based on calibration using the ruler and an observer distance of 1 m. The measured luminance in Figure 10b is 138.3 cd/m^2^ and the angular size of the green “G” is 4.6°. Based on Equation (6), the calculated modulation under these conditions is 0.0039, which is lower than the measured result of 0.0109 shown in Figure 11.

**Figure 12:**
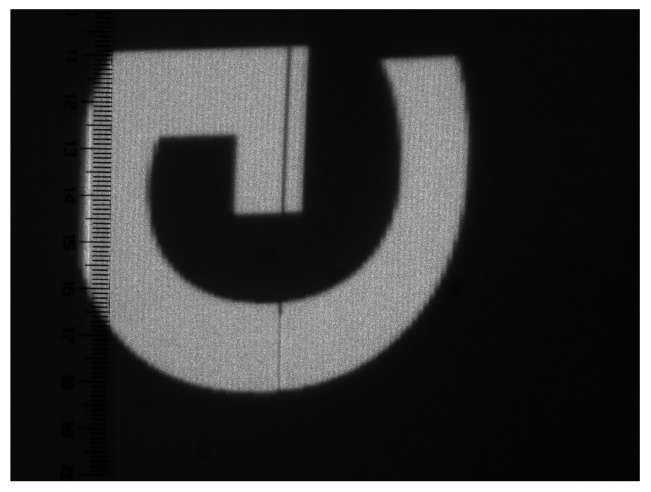
Image combining two-frame scanning trajectories captured by the CCD.

In this study, the limited sampling rate (100 kHz) of the control system’s feedback signal made it difficult to accurately characterize variations in the micromirror scanning trajectory by comparing the vertical feedback signals from each frame. Instead, we directly observed the changes in the scanning trajectory by continuously capturing multiple image frames. Each frame had an exposure time of 16 ms, and the total acquisition time was 1 s, during which 63 images were captured in total. Since the exposure time could not be perfectly synchronized with the scanning period, some of the captured images contain a combination of different regions from two-frame scanning trajectories, which results in a noticeable dark band, as shown in Figure 12. Among the 63 images, 4 fell into this category and were therefore discarded from the analysis.

**Figure 13:**
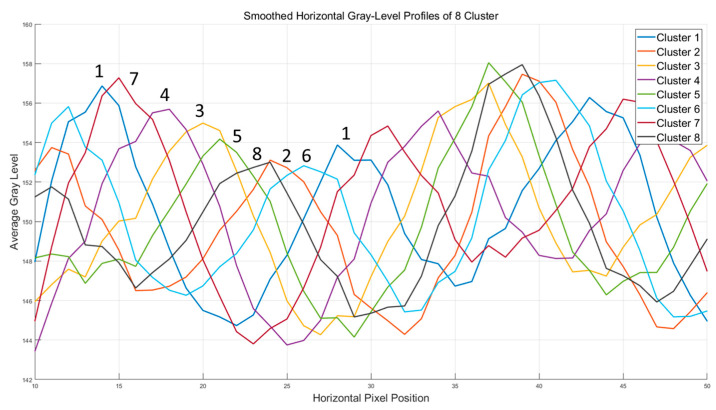
Smoothed horizontal gray-level profiles of 8 clustered trajectories.

We compared the remaining 59 acquired images using MATLAB R2018a, performed clustering analysis, and manually reviewed the results. The analysis results of the images are shown in Figure 13. As a result, the scanning trajectories were grouped into eight clusters. It can be observed that seven new scanning trajectories (2-8) were added across two adjacent rows of a trajectory (1-1). During the analysis, we mainly focused on the differences between scanning trajectories. For two trajectories that are offset by one horizontal scanning period—as shown in Figure 7—we grouped them into the same cluster. Due to the camera’s resolution limitations, the gap between adjacent rows is approximately 16 pixels. After inserting seven trajectories, the average gap between two adjacent rows reduces to only 2 pixels. To better characterize trajectory changes, we did not solely analyze the pixel information from a single column perpendicular to the bands. Instead, we selected a region and performed a more comprehensive analysis. During data processing, a simple smoothing technique was applied to reduce the effects of speckle and other factors.

The classification of all 63 images is shown in Table 1**.** The number indicates the sequential order in which the images were captured. Table 1 and Figure 13 show that the variations in scanning trajectories occur in a random and unstructured manner.

**Figure 14:**
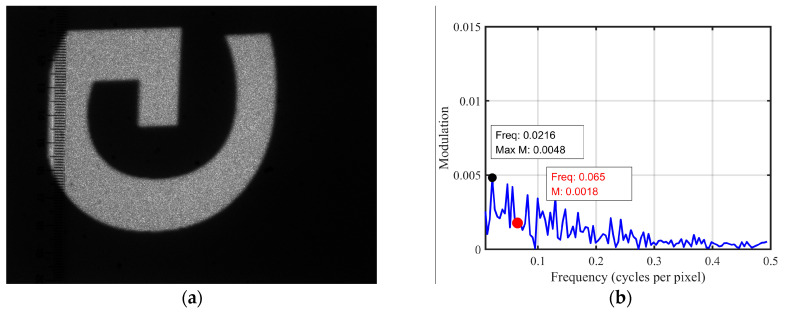
(**a**) Combined trajectory image; (**b**) spatial frequency profiles of the combined trajectory within the same region as the single-frame scanning trajectory.

The combined trajectory was captured with an increased exposure time of 133,333 µs, as shown in Figure 14a. Figure 14b shows the results of the FFT analysis, performed on the same region as in Figure 10b. At a frequency of 0.065 (Figure 10), the modulation depth has decreased to 0.0006, which can be considered nearly zero, indicating that both the banding artifacts and non-uniform distribution have been mitigated.

However, at this point, other noise appears with a maximum value of 0.0043, still lower than the initial value of 0.0109. This could be attributed to the relatively long exposure time used when capturing the combined trajectory during the experiment. To compensate, we slightly reduced the camera’s aperture to ensure that the overall grayscale of the combined trajectory image matches that of the single trajectory image. As a result, the overall grayscale of the image is slightly lower than that of the single frame, meaning some pixels have lower gray levels. However, many pixels still maintain high grayscale values due to laser speckle (an optical interference phenomenon), leading to this increase in modulation.

## 5. Conclusions

Banding artifacts and non-uniform spacing are defects inherent to raster bidirectional scanning displays. In this paper, we address these issues by adjusting the retrace time to modify the scanning trajectory. The interleaving of different scanning trajectories increases the scanning density well beyond the image resolution. Due to characteristics of human vision, the variation in retrace time must be randomized. In practical control, this variation is implemented as integer multiples of 1/8 of the fast-axis period. The pixel information of the scanning image is dynamically adjusted according to the changes in trajectory. When the trajectory deviates from the ideal pixel positions, interpolation algorithms are used to compute the new pixel color values. These approaches effectively reduce visual artifacts and support the broader adoption of scanning projection technology.

In addition, this method relies on the accurate angle feedback signal from the MEMS mirror. In the control system, it is necessary to detect in real time the resonance frequency of the mirror based on the angle feedback signal and adjust the control according to the latest resonance frequency.

## Figures and Tables

**Table 1:** Classification of scanning trajectories.

	Cluster 1	Cluster 2	Cluster 3	Cluster 4	Cluster 5	Cluster 6	Cluster 7	Cluster 8	Discarded
Number	07, 08, 17, 26, 27, 36, 45, 54	05, 12, 22, 30, 31, 39, 41, 42, 51, 60	15, 24, 33, 43, 62	11, 21, 40, 49, 50, 59	09, 16, 25, 34, 35, 44, 55, 63	01, 02, 03, 18, 28, 37, 46, 48, 57, 58	04, 29, 38, 47, 56	06, 13, 14, 23, 32, 52, 61	10, 19, 20, 53

## Data Availability

The original contributions presented in this study are included in the article. Further inquiries can be directed to the corresponding authors.
